# Gender essentialism in transgender and cisgender children

**DOI:** 10.1371/journal.pone.0224321

**Published:** 2019-11-13

**Authors:** Selin Gülgöz, Madeleine DeMeules, Susan A. Gelman, Kristina R. Olson

**Affiliations:** 1 University of Washington, Seattle, Washington, United States of America; 2 University of Michigan, Ann Arbor, Michigan, United States of America; University of Maryland, UNITED STATES

## Abstract

Children, across cultures, show an early-emerging tendency to essentialize gender, viewing gender as inborn and predictive of stereotypical preferences. However, research has been limited to children whose own gender experience is largely consistent with the assumptions of gender essentialism. In contrast, transgender children have gender identities (and related stereotypical preferences) that differ from their sex assigned at birth, which therefore appear to challenge an essentialist view of gender. In the current study, we examined the degree to which transgender children (*N* = 97, 3–11 years) view a child’s sex at birth as predictive of their later gender-typed preferences. Additionally, we recruited two comparison groups: cisgender siblings of transgender participants (*N* = 59) and cisgender, age- and gender-matched controls (*N* = 90). In an adapted switched-at-birth paradigm, participants in all groups believed that a child’s sex at birth would predict their later gender-typed preferences; participants were especially likely to think so when the target character was reared in a socialization environment that aligned with the target’s own gender, rather than one where the socialization environment aligned with a different gender. Whereas cisgender participants showed a decline in essentialism with age, transgender children did not show any age-related changes in their beliefs. The current findings are the first to show that transgender and cisgender children, despite differences in gender experiences, might similarly essentialize gender. However, these findings also raise questions about how different participant groups might interpret measures differently.

## Introduction

Gender essentialism refers to the belief that gender is a discrete and dichotomous social category (i.e., one can be either a girl or a boy, but not both, nor somewhere in between), and that gender is inborn, biologically determined, immutable, and informative of categorical properties [1–2]. Gender is perhaps the earliest emerging and most salient social category [3], and gender essentialism is believed to be a basic feature of human social categorization, with children across cultures showing early essentialism of gender [4–10]. The current study examines gender essentialism in *transgender children* (defined in this study as children who socially transition at an early age to live and present as a gender that differs from the gender typically associated with the sex assigned to them at birth). In contrast to the gender identity of cisgender children (i.e., children whose gender identities are aligned with their assigned sex), transgender children’s gender identity challenges several tenets of gender essentialism (e.g., that gender is biologically determined). However, to this point, no research has been conducted on whether transgender children and cisgender children differ in gender essentialist beliefs, perhaps in part because relatively few children in our society identify as transgender [11].

### Gender essentialism in cisgender children

Previous research has documented strong gender essentialism among cisgender children, especially in early childhood. For example, by 3 to 5 years of age, children expect that gender will stay the same throughout the lifespan (e.g., a girl will grow up to be a mom; [12–14]), even when reasoning about a child who is raised solely among other-gender individuals (e.g., believing that a girl who is raised entirely among boys and men will still grow up to show properties stereotypically associated with girls; [9–10]). Additionally, by four years old, cisgender children believe that even if a girl looks like a boy, as long as she is categorized as a girl, she will share more properties (e.g., preferences in novel activities) with other girls than boys [6, 15]. By age five, cisgender children reject the possibility that a girl and a boy might be the same kind of person, viewing these categories as discrete, natural, and not determined by convention [5, 8]. And when six-year-olds are asked why a girl might want to play dress-up rather than baseball, they give the essentialist explanation that girls were born this way [10].

Among previous research examining cisgender children’s gender essentialism, at least one study has examined cisgender children’s essentialism when they are asked to reason about transgender targets. When they heard descriptions of a child who identified and presented as a gender different from what they were assigned at birth, about half of 5- to 11-year-old cisgender participants consistently believed that the target child should be categorized as the gender associated with their sex at birth [16]. Thus, from a young age, children might view gender as inborn, biologically based, stable, and predictive of other nonobvious properties, even in cases where outward environmental influences might provide contradictory information.

### Transgender children’s gender concepts

Although transgender children have been increasingly visible in mainstream U.S. media in recent years [17–19], little empirical research has documented the development of their gender concepts. The few studies that have examined socially-transitioned transgender children’s gender development have demonstrated similarities to their same-gender peers (e.g., in gender identities and preferences; [20–22]). However, given that transgender children’s early experiences with gender differ from those of cisgender children, differences in their gender concepts might also be expected.

Because cisgender children have overlapping sex and gender identities (i.e., a cisgender male—sex—also most likely identifies as a boy—gender identity), and people around them tend to treat them in ways consistent with their gender identity, they may be more likely to view gender as inborn, biologically based, stable, and informative of gender-typed preferences. Transgender children’s experiences with gender are unique because prior to transitioning, they live and are treated as one gender (i.e., the gender aligning with their assigned sex) while they identify as a different gender on the inside. Once they socially transition to live and present as their self-identified gender (changing pronouns, appearance, and name; [23]), transgender children are treated by others as no longer conforming to the gender aligned with their assigned sex. The discrepancies in their early gender presentation and identification, and the switch they experience in how others categorize their gender might lead transgender children to develop different beliefs regarding the inborn, biological, and stable nature of gender. Moreover, transgender children, by virtue of having a gender identity that does not align with their assigned sex, might have more flexible views of how predictive one’s assigned sex is of a person’s gender-typed preferences.

Although most findings to date on socially-transitioned transgender children’s gender *development* have shown similarities to same-gender cisgender peers [20–22], a few key differences have also been found in transgender children’s *beliefs* about gender. For example, when reasoning about others’ gender identities, 3- to 5-year-old transgender participants and their siblings were more likely than cisgender controls to report that gender identities could change [20]. This might indicate that transgender children are less likely than cisgender children to essentialize gender, as an essentialist outlook assumes that gender is immutable.

In a related study, Olson and Enright [24] found that 6- to 8-year-old socially transitioned transgender participants and their cisgender siblings showed greater tolerance for others to express gender nonconformity compared to cisgender controls. This suggests that transgender children and their cisgender siblings might not view one’s gender to be as informative of preferences and behaviors, relative to children who exhibit gender development typically seen in cisgender children.

Together, these findings indicate that having transgender identities (in the case of transgender participants) or being exposed to them (in the case of cisgender siblings) might influence children’s essentialist beliefs, particularly about the inborn nature, stability, and informativeness of gender. However, perhaps because these studies are few and limited in sample size, findings have been mixed regarding whether there are differences between transgender and cisgender children in their gender stereotyping [20, 24]. These contrasting results raise questions about whether transgender and cisgender children might resemble one another in their beliefs about sex at birth being predictive of stereotypical properties even when environmental conditions provide contradictory evidence.

### The current research

The current research provides the first investigation of how a child’s own gender identity expression (as transgender or cisgender) relates to their gender essentialism, specifically their tendency to use information about a target’s sex at birth to infer later gender-typed preferences. For this purpose, we recruited transgender children and age- and gender-matched cisgender controls, and examined whether these groups differed in their gender essentialism. Moreover, when available, we recruited a second control group composed of cisgender siblings of transgender participants, to account for the socialization environment that might be unique to transgender children’s households, as well as the relative roles of *having* a gender identity that contradicted an essentialist view of gender vs. *exposure* to such an identity.

We used a classic measure of gender essentialism (a switched-at-birth task), versions of which have been frequently used to measure individuals’ essentialist reasoning about gender as well as other categories [9–10, 25–27]. In the key parts of this study, participants were told about a baby (e.g., a baby boy) who at birth was taken to an island where the child was raised, interacting only with children and adults of a different gender (e.g., girls and women). Participants were then asked a series of questions regarding the child’s future gender-stereotyped preferences (e.g., whether the child would like to wear dresses, whether the child would like to play football). The task measured children’s likelihood of viewing the character’s sex at birth as informative of their later stereotypical properties even when raised in a rearing environment aligned with a different gender (e.g., thinking that a boy wants to play football, regardless of environmental influences), which would indicate essentialist reasoning regarding gender. Further, we contrasted this case with a case in which the target character is reared by adults of the same gender, in which the socialization environment and the child’s sex at birth would be overlapping.

Given transgender children’s unique experiences with their own gender identities, and based on findings of previous research described above [20,24], we predicted that transgender children and their cisgender siblings would show lower rates of essentialism than cisgender controls. That is, if transgender children and their siblings do not believe that gender is a direct result of biological sex, they also might not find sex at birth as predictive of later gender-typed preferences. However, being the first study of transgender children’s gender essentialism, the current research is largely exploratory; given mixed findings from previous research examining transgender children’s beliefs about gender, alternative patterns of results were certainly also plausible.

In the current research, we also explore whether transgender children’s gender essentialism changes across development. Some prior research with cisgender children has found that children are initially robustly essentialist about gender, and are increasingly influenced by environmental factors as they get older [8]. Thus, one possibility in the current research is that children’s own unique gender experiences might not influence their essentialist reasoning until later in development. Moreover, any differences in how transgender and cisgender children essentialize gender might appear only among older children. For this purpose, the current study explores gender essentialism in a wide age range (3- to 11-year-olds).

## Method

### Participants

Children in this study participated in a larger, longitudinal project on gender development among transgender children. The current study included three groups of participants: (1) socially transitioned transgender children (henceforth, transgender), (2) cisgender siblings of transgender children (henceforth, siblings), and (3) unrelated cisgender participants who were age- and gender-matched to each transgender participant (henceforth, unrelated controls). The current study was approved by the University of Washington Institutional Review Board (approval #00001527). All participation was conducted only once parents had provided written consent, and children had provided verbal (ages 3 to 8 years) or verbal and written assent (ages 9 to 11 years). Participants received a small toy and $10 for incentive. Recruitment procedures for each group of participants are described in further detail below. Because this task was part of a larger longitudinal study, participants received additional measures at time of testing. However, the other measures and associated findings are described in other papers, because they are not related to essentialism.

#### Transgender participants

Transgender participants were 97 3- to 11-year-olds (see Table 1 for participant demographics). Participants in this group had all socially transitioned (i.e., were living and presenting as the gender contrasting with their assigned sex and using the associated pronouns; see [20, 24]) at the time of data collection. Fifteen additional transgender participants began the task but did not complete it. Because partial data could not be analyzed (i.e., analyses required answers for both the other-sex and sex-matched rearing conditions), their data are excluded from the current analyses.

**Table 1:** Participant demographics.

		Transgender	Cisgender Siblings	Cisgender Controls
Participants *N*		97	59	90
Age *M* (*SD*)		7.89 (2.08) years	7.90 (2.09) years	7.89 (2.03) years
Gender		63 girls, 34 boys	20 girls, 39 boys	59 girls, 31 boys
Ethnicity				
	White/European	68%	73%	73%
	Hispanic/Latino	-	5%	1%
	Black/African	1%	-	-
	Asian	5%	2%	3%
	Multiethnic	25%	17%	21%
Income				
	Less than $25,000/year	4%	5%	1%
	$25,001–50,000/year	5%	10%	1%
	$50,001–75,000/year	18%	14%	9%
	$75,001–125,000/year	37%	39%	36%
	Greater than $125,000/year	33%	29%	46%
	Missing	3%	3%	7%
Parent Political Orientation^a^ *M* (*SD*)		1.58 (0.77)	1.70 (1.00)	2.27 (1.23)

^a^ Political orientation scores range from 1 (very liberal) to 7 (very conservative).

Transgender participants were recruited through national online and in-person support groups and conferences for families with transgender children, via word-of-mouth, and in response to media coverage of the larger project. Experimenters traveled throughout the U.S. to meet transgender participants and their families, conducting testing sessions in their homes or at conferences or in private spaces in public buildings (e.g., churches, libraries, etc.); in the case of participants local to the primary researchers, participants were tested in a developmental psychology lab. Although pilot data on the same task were collected from a group of gender-nonconforming participants as well (i.e., participants who have not socially transitioned but show preferences that are more closely aligned with a gender contrasting with their assigned sex), only a small number of participants within this group (*n* = 23) completed this task so their data (and data from their siblings and unrelated controls) are excluded from the current paper.

#### Siblings

Cisgender siblings were 59 4- to 11-year-olds (see Table 1 for participant demographics). In cases where there were multiple siblings eligible to participate (i.e., in the right age range), the sibling closest in age to the transgender child participated. Cisgender siblings were recruited and tested at the same time as their transgender siblings, using the same recruitment techniques.

#### Unrelated controls

Unrelated controls were matched to transgender participants on age (to be within 4 months of the transgender children’s age at time of testing) and gender (to match transgender children’s gender identity). For example, a 6-year-old transgender girl (i.e., a child assigned male at birth who had socially transitioned to present as a girl) would be matched to a 6-year-old cisgender girl. This is the same matching protocol used by Olson and colleagues [21] and other papers. The matching protocol for controls as well as which controls do and do not get included in analyses is posted in our pre-established lab protocol (https://osf.io/ypzg9/). According to the rules in this protocol, the final unrelated control group included 90 3- to 11-year-olds (see Table 1 for participant demographics). Unrelated controls were recruited through the child participant database of a university in the Pacific Northwest, U.S. During recruitment, families of participants were informed that their child was being recruited for a longitudinal study on gender diversity. As per the protocol, data from an additional 7 participants who were matched to transgender participants included above but who completed only part of the task, were not included in the analyses and the matched controls for the 14 transgender participants who did not complete the task were not included in the main analyses(one matched control also did not complete the task). In addition, data from 11 additional control participants were accidentally collected due to experimenter error.

### Measure and procedure

Participants received a modified version of the Island Task [9–10]. Each participant heard a total of 4 vignettes that described a baby boy or girl raised in sex-matched or other-sex rearing environments. For example, on one of the sex-matched trials, participants heard about a baby girl who was raised on an island inhabited only by girls and women, whereas in the other-sex condition, they heard about a baby girl who was raised on an island inhabited only by boys and men. Each participant heard the items in the same set order: boy raised among boys and men, girl raised among boys and men, girl raised among girls and women, boy raised among girls and women. This set order was used for logistical reasons having to do with this task being embedded within a larger protocol of other tasks unrelated to the current paper, each of which was in a fixed order for all participants. After hearing about each child, participants were asked 4 questions about each target child’s future preferences about two properties stereotypical of girls (wear dresses, play with dolls), and two properties stereotypical of boys (have short hair, play football). In line with past use of this task [10], trials were scored such that participants received 1 point if they expected the target children to show properties stereotypical of the target’s sex at birth or not to show properties stereotypical of the other sex at birth. If participants expected the target children to show properties stereotypical of the other sex (i.e., on other-sex rearing trials, the sex of the people in their environment) or to fail to show properties stereotypical of the target’s sex at birth, they received 0 points. On the sex-matched rearing trials, participants’ beliefs in either socialization or essentialism would lead to higher scores, while counter-stereotypical inferences would lead to lower scores. On the other-sex rearing trials, socialization was pitted against essentialism, such that essentialist inferences would lead to higher scores, and socialization-based inferences would lead to lower scores.

## Results

As a preliminary test, we wanted to ensure that the three groups of participants were equivalent in age. We conducted a one-way ANOVA of participant group (3: transgender, siblings, controls) on age. Results showed no significant differences in age as a function of participant group, *F*(2,243) = 0.01, *p* = .999, *η*_*p*_^2^ < .01

Given the categorical nature of the response choices, we used Generalized Estimating Equations (GEE) to assess the extent to which groups differed in their gender essentialism (see Table 2 for descriptive statistics). We conducted a 3 (participant group: transgender, siblings, controls) x 2 (trial type: sex-matched rearing, other-sex rearing) x 2 (target gender: boy, girl) binomial logistic regression on essentialism scores on each trial, with participant age included as a continuous covariate, using an exchangeable covariance matrix, which yields Wald *χ*^2^ values as indicators of main effects and interactions. We found a significant effect of trial type, *χ*^2^ (1) = 13.33, *p* < .001, indicating that participants made more inferences based on the target’s sex at birth when the target was raised among people who shared their sex (*M* = .81, *SE* = .02, 95% CI .78, .84) than when the target was raised among people of the other sex (*M* = .65, *SE* = .02, 95% CI .61, .69). There were no significant effects of participant group, *χ*^2^ (2) = 3.92, *p* = .141, age, *χ*^2^ (1) = 0.05, *p* = .818, or target gender, *χ*^2^ (1) = 2.59, *p* = .108, and no significant interactions of participant group x age, *χ*^2^ (2) = 2.49, *p* = .287, participant group x target gender, *χ*^2^ (2) = 1.15, *p* = .564, target gender x trial type, *χ*^2^ (2) = 0.15, *p* = .698, target gender x age, *χ*^2^ (1) = 1.65, *p* = . 200, participant group x target gender x trial type, *χ*^2^ (2) = 0.03, *p* = .984, participant group x target gender x age, *χ*^2^ (2) = 1.49, *p* = .476, target gender x trial type x age, *χ*^2^ (1) = 0.80, *p* = .371, or participant group x target gender x trial type x age, *χ*^2^ (2) = 0.35, *p* = .840.

**Table 2:** Descriptive statistics for each participant group for each trial type.

Participant group	Rearing type					
	Same sex as target			Different sex from target		
	M	SD	95% CI	M	SD	95% CI
Transgender	0.78	0.03	.73 - .83	0.64	0.03	.58 - .70
Control	0.86	0.02	.82 - .89	0.69	0.03	.63 - .75
Sibling	0.80	0.04	.72 - .87	0.62	0.04	.55 - .70

Note. The 95% Confidence Intervals did not include .50 for any means, suggesting that participants in all groups used sex at birth to in making inferences about future preferences in both types of rearing environments.

**Fig 1:**
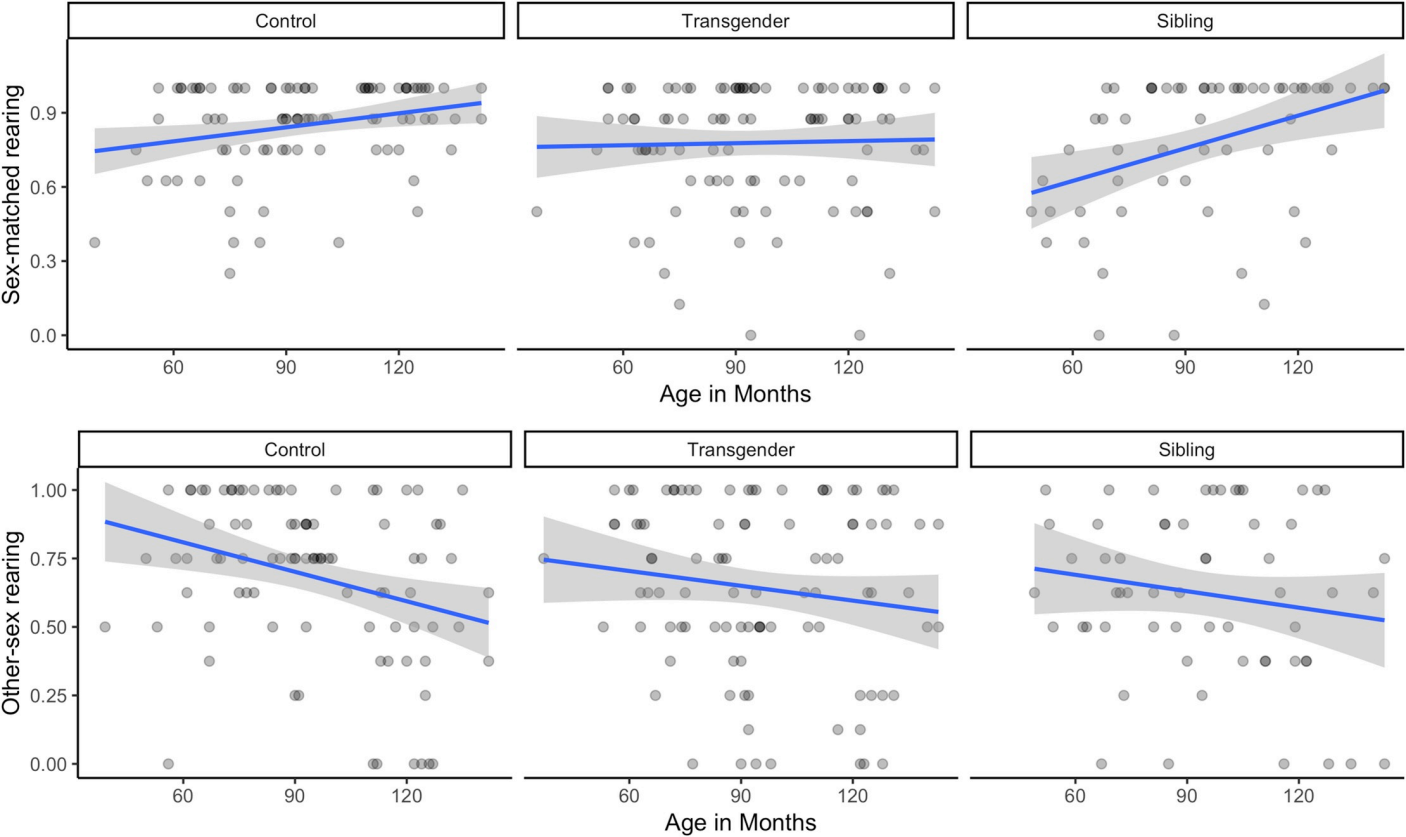
Scatterplots depicting correlations between age and essentialist inferences on sex-matched and other-sex rearing trials for each participant group.

However, there was a significant trial type x participant group interaction, *χ*^2^ (2) = 6.85, *p* = .033, and a significant trial type x age interaction, *χ*^2^ (1) = 28.93, *p* < .001. These two-way interactions were further subsumed under a three-way trial type x participant group x age interaction, *χ*^2^ (2) = 7.35, *p* = .025. To understand this three-way interaction further, we conducted correlation analyses between essentialism and age on each type of trial, separately for each participant group (see Fig 1). We found that older control participants made more inferences based on sex at birth on sex-matched rearing trials, *r*(90) = .25, *p* = .016, and fewer inferences based on sex on other-sex rearing trials, *r*(90) = -.30, *p* = .004, a pattern demonstrating a developmental increase in the belief that the sex of parents influences a child’s gendered behaviors. In contrast, for transgender participants, there were no significant correlations with age (sex-matched rearing trials: *r*(97) = .03, *p* = .779; other-sex rearing trials: *r*(97) = -.14, *p* = .158). Siblings made more sex-based inferences as they grew older on sex-matched trials, *r*(59) = .39, *p* = .002, but not on other-sex trials, *r*(59) = -.16, *p* = .214; however, correlations conducted with siblings should be interpreted with caution, given the low sample size.

The data and analysis codes are available online at https://osf.io/3rmf9/.

## Discussion

When asked to make inferences about a baby’s future gender-typed preferences, transgender participants, their cisgender siblings, and cisgender controls all reliably used the target’s sex at birth to predict whether that target character would show feminine-typed or masculine-typed preferences in the future. Importantly, this was true both when targets were raised by parents of the same sex as the child and when targets were raised by parents of a different sex than the child—though across groups, participants also believed that children raised by parents who shared their sex would have stronger gender-typed preferences than children raised by parents who did not share their sex.

On sex-matched rearing trials, the target child’s sex at birth aligned with the socialization environment. As alluded to above, these cases might best be thought of as a baseline measure of children’s stereotyping, and in this case our findings show that, in the absence of other information, both transgender and cisgender children made stereotypical inferences. On other-sex trials, which assessed participants’ gender essentialism by pitting a target’s sex at birth against the target’s rearing environment, our results further indicate that both transgender and cisgender participants essentialized gender. These findings are consistent with previous literature on cisgender children’s gender essentialism [10]. However, we also observed that transgender children presume the endorsement of stereotypical preferences by others. This finding emerged despite previous research showing that transgender participants, and siblings who have been in contact with transgender people, might believe that children can have counter-stereotypical preferences [24], and transgender children have sometimes been shown to endorse gender stereotypes less than their cisgender peers [24], though see [20].

The degree to which participants made sex-based inferences varied by trial type: participants made more sex-based inferences when the character was raised by those who matched their sex (e.g., a boy raised by males) than when raised by those who differed in their sex (e.g., a boy raised by females). This is expected, because in sex-matched trials the target’s sex at birth and the gender of the socialization environment are aligned. Though participants viewed sex at birth as informative of later preferences even in the other-sex rearing trials, that they did so to a lesser extent suggests they believe that socialization plays at least a partial role in determining children’s gender-stereotyped preferences.

Although we did not find an overall robust change in children’s responses with age, there were variations in response patterns for specific participant groups that differed by trial type. As cisgender controls grew older, they made more stereotypical inferences on sex-matched trials, and fewer stereotypical inferences on other-sex trials. In combination, these effects suggest that cisgender controls increasingly viewed socialization as predictive of children’s gender-stereotypical preferences. This is consistent with previous research showing that as cisgender children grow older, they show lower levels of essentialist reasoning about gender, and begin to view gender as more open to environmental influence [8, 10]. Cisgender siblings showed a similar pattern: their stereotypical inferences on sex-matched trials also increased with age (no change was seen in other-sex trials). Though, this effect should be interpreted with caution given the smaller sample size. Transgender children, however, did not show any age-related changes in their responses on either sex-matched or other-sex trials, which could suggest a developmental pattern that contrasts with that of cisgender children, among other possibilities. It is unclear why transgender children might not increasingly believe in the role of socialization as they get older. Because this is the first study known to examine transgender children’s essentialist reasoning, and due to limitations highlighted below, these results should be interpreted with caution until the study is replicated.

### Theoretical implications

Researchers have speculated about the origins of essentialist thinking. The current findings suggest that children might develop an essentialist view of gender early in life, even if their own gender experiences are at odds with certain aspects of essentialism. For example, our data show that even a transgender child who does not show preferences in activities that are stereotypically associated with their sex assigned at birth might still expect others to show stereotypical properties consistent with their sex assigned at birth. As such, these findings demonstrate the prominence of essentialist thinking in young children’s social reasoning, and suggest that children essentialize categories even when their own identities cross category boundaries. These findings provide support for the idea that essentialist thinking has foundations in basic cognitive processes [1], as evidenced by transgender children’s reliance on stereotypical information, rather than reflection on possible individuating differences, in the face of minimal cues for making inferences about others. That transgender children rely on broader social influence rather than own personal experience in making inferences about others demonstrates the power of categorical thinking in children’s early reasoning.

Additionally, the current work suggests that transgender children might share other children’s intuitions that gender, even their own gender, is inborn and biologically determined. That is, they might believe that what led to their gender identity and expression was some aspect of their biology, even though it did not align with their sex assigned at birth. Thus, transgender children might be as essentialist as cisgender children—they might just ascribe gender identity, rather than sex assigned at birth, as an essentialized attribute.

### Limitations

Though novel in its inclusion of transgender children, the current sample is still limited in several ways. The transgender children in this study are unique: they have socially transitioned at a young age, and they come from families interested in research participation. Therefore, caution is warranted when generalizing these findings to a larger population.

In this study, we introduced the target’s gender by telling participants that the character was either a boy or a girl. This approach is commonly used in the literature and was deliberately selected for the current work. Given the relative scarcity of research to date on transgender children’s gender cognition, a good starting point was to use established, well-documented measures (also see [20]). However, one limitation of this approach (in our study and in the field) is that the experimenters do not explain whether “boy” and “girl” refer to the child’s *sex* as male/female (i.e., what the child was assigned at birth based on genitals) or the child’s *gender identity* as boy/girl (i.e., what the child feels they are). Throughout this paper, we have referred to this assignment as the child’s sex, but we do not know that our participants made that inference or, more importantly, if children’s inferences could have varied by the group they were in. For example, transgender participants might interpret “boy” as referring to a child living and presenting as a boy and think that that will lead him to have short hair, whereas cisgender children might interpret “boy” as referring to a child who was born as a male and think that that will lead him to have short hair. This limitation of the current measure was made particularly apparent with the inclusion of transgender children in the current study, demonstrating the need for diversity in recruited samples. Future research with more precise measures that clarify this point, as well as more qualitative investigations assessing children’s open-ended explanations regarding their gender-related inferences, are needed to understand whether transgender and cisgender children are using the same logic in reasoning about the role of gender in determining later stereotypical properties.

Finally, the lack of differences between transgender and cisgender children in the current work might be a reflection of children’s binary identities. In the current samples, transgender and cisgender children used binary gender pronouns. Future work recruiting children with nonbinary identities might investigate the role of having a categorical identity on essentialist reasoning.

## Conclusion

The current study was the first to examine transgender children’s beliefs about the inductive potential of one’s sex at birth. Regardless of differing early experiences with gender, findings from the current study suggest that transgender and cisgender children do not appear to differ in how they reason about this aspect of gender. That is, when asked to make inferences about a child’s preferences, both transgender and cisgender children relied on stereotypical information. Further, even though transgender children themselves tend not to have preferences that align with their sex at birth [21], they think that others are more likely than not to have preferences aligned with their sex at birth. This finding may indicate that transgender children are aware that being transgender itself is quite rare, or the finding could reflect a different interpretation of the stimuli, where transgender children believe that we were assessing gender rather than sex. These data provide an initial foray into understanding the ways in which transgender children do or do not differ from other children in thinking about gender essentialism.
